# Proceedings of the Cincinnati Academy of Medicine

**Published:** 1879-10

**Authors:** 


### PROCEEDINGS OF THE CINCINNATI ACADEMY OF MEDICINE.

The regular essay for the evening was read by Dr. Forchheimer, subject scarlet fever.

Dr. Cleveland agreed with the essayist that the course of the disease varied much in different cases. The treatment, as a rule, was not very satisfactory. Malignant cases would die no matter what was done, while mild ones had a tendency to recover. The speaker had no stereotyped method, but, as a rule, treated the symptoms as they arose. He thought that he had seen good results from salicylic acid. It increased the flow of urine, and the local effects on the throat were good. To keep the fever within bounds, greasing the body and luke warm baths were the methods he usually resorted to.

He had been called to a case where a child died of malignant scarlet fever. The mother was in the ninth month of pregnancy, and she was delivered on the day her child was buried. In twenty four hours after delivery, she had a fever 102—103°. No abdominal tenderness. In a short time the scarlatinous eruption appeared. The case progressed favorably. No septicæmic trouble appeared. There was a small amount of albumen in the urine which soon disappeared, and the patient recovered.

Dr. Whittaker said he had never seen a case in the newly born. The disease usually attacks children between the years of three and five. The fact that the eruption of scarlet fever may be hæmorrhagic, is an evidence that it should be classed with other acute infectious diseases, as small pox, typhoid, etc. The worst and most malignant cases the speaker had ever seen were those without an eruption. Anderson says that the most malignant form of scarlet fever may kill in six hours before the eruption has had time to appear.

As regards treatment, the most important thing to guard against is disease of the kidney. It is more important to watch the urine, than the pulse or temperature. Nor must we think that because the attack may be mild nephritis is not likely to occur. For kidney complication is an attendant more often of the mild than of the severe cases. There is no especial gravity when we find in the urine only albumen and epithelium, but when casts are present, the danger is increased, because the casts show that the tubules of the kidney are choked up. As to treatment, he had been in the habit of giving digitalis early. The only preparation that he had found reliable was infusion of the first English leaves. It seemed to have occured that since he had been giving this remedy early and in round doses, the nephritis as a sequellæ of scarlet fever, had become rarer in his practice than it was before he began to prescribe it.

Although the disease is very malignant and contagious, it travels from place to place very slowly. It was first observed in this country in 1729, but it was not until 1825 that it was seen in South America, and it appeared in Australia in 1848.

Dr. Zinke said that the disease had never left the German quarter of the city, and that within the last two years he had seen about fifty cases. His usual treatment was the tincture of aconite in doses of ½ drop and 1 drop every hour and with very good results. Where aconine has not the desired effect in reducing the temperature, he had recourse to quinine 1 gr. per hour. The great danger, in his opinion, in these cases was the catarrhal affection of the throat extending to the trachea and lungs. For the catarrh and membranous affection of the throat he had been using with most happy results inhalations of quinine or cinchonidia. Since he had used them he had seen no cases die on account of the complication with the throat or lungs. The speaker had never seen in his practice a fatal case of post scarlatinous nephritis. All patients where the complication was present did well under digitalis. He had never used pilocarpine.

Dr. Forchheimer said that most text-books put the appearance of nephritis too late, because the œdema, as a rule, calls the physician's attention to the candition of the kidneys instead of the presence of albumen in the urine doing so. He had often been enabled to prophecy the occurrence of œdema, and to put the child under treatment. His invariable rule was to examine the urine at the beginning of the 2d week of the disease. Had seen albumen as early as the seventh day. At first the amount of albumen present in the urine is very slight. It is necessary to diagnosticate the nephritis early, especially if we expect to derive any good from the exhibition of digitalis, for it takes from 10—24 hours before the physiological effects of the drug are manifest.

Dr. Thad. A. Reamy said he could not agree with his colleagues, Drs. Whittaker, and Forchheimer, that it is far more important to test the urine for albumen in cases of scarlet fever, than to mark the pulse rate and the temperature. No judicious practicioner will omit at the proper time to test for albumen, and so far as possible to ward off the grave leison that its presence threatens, parenchymatous nephritis. And among the agents to be employed under such circumstances, certainly digitalis stands pre-eminent; but when we consider first, the conditions which often precede, and indeed, induce the nephritis, and secondly, the modes by which digitalis must act in accomplishing the kidney relief, we are not justified in the statement that temperature and pulse are of less importance than the examination of the urine. It is probably true that digitalis has some direct duretic influence upon the kidney, but it is not this influence which commends it in these cases as an agent of such immense value. It is the power of digitalis to stimulate the cardiac ganglia, and its unquestioned action upon the vaso-motor centre, as shown by Fothergill, Traub and Bartholow, thus increasing the arterial tension and relieving venous congestion, that enables it to relieve the renal veins, and in the forcible language of Prof. Whittaker, "flush out the kidneys."

The cardiac failure in these cases, demanding the influence of digitalis or some such agent, may be due to the overwhelming influence of the scarlatinal poison upon the nervous centres, or to the impaired nutrition of the heart, which in such cases so rapidly ensue, or to the impaired power of the heart, resulting from the influence of intense temperature upon its muscular structure. Indeed, these states may in some cases, be considered as in the logical order of cause and effect.

As to temperature, I have never yet seen during a clinical experience of many years, and with many cases, a single recovery where the temperature continued as high as 105.5° F., during any consecutive 3 days or the first week. I have seen recoveries after a temperature of 107° in two or three instances, but this range was maintained but a few hours, then falling below 105. I have never seen a case recover where the temperature ranged as high as 105.5° for 24 consecutive hours.

It is not very unusual for cases to recover when the temperature has reached 106° or even above, but has within a few hours fallen to 104° or below. In fact, oscillation of temperature with some show of uniformity as to time within the 24 hours, even though a very high range be reached during the pyrexia, is more favorable by far than a steady range for 24 hours of even 104 to 104.5. A continued high temperature in this disease will invariably be associated with a failing pulse volume. No matter whether we accept the statements of Sekerman and Heidenham (that the temperature and arterial blood pressure vary inversely) or not; it cannot be denied that digitalis acts most successfully as an antipyretic in cases of high fever associated with heart failure.

Now, in cases of scarlet fever, nothing so certainly reduces the high temperature of the first week as sponging off limbs and body every half hour or hour with cold or tepid water. This is in my experience preferable to a plunge bath. And this method secures as effectually as possible the action of the skin, thus protecting the kidney in a two fold manner.

I would insist therefore upon watching temperature and pulse, thereby putting the practioner in possession of facts which may enable him to ward off and effectually prevent, the approach of the serious complications to which the able essay and the remarks upon it by my friends give such prominence.

My experience with scarlet fever has been singular. For 4 years I did not loose a single case, but last year I encountered it in so virulent a form that I lost 3 children in one family; two died in the same night. Not only do certain families bear the disease badly, but during the same epidemic and at the same time, certain squares of the city show singular mortality. There is much yet to learn as to range of the infecting power, isolation, propholaxy by quinine, belladonna, etc.

Dr. Whittaker said the effect of digitalis was to strengthen the heart, and secondarily to effect the kidneys by forcing more blood through, then causing an increased flow of urine, and hence to flush out the tubules. The two points to be observed in regard to digitalis was first, to have a good infusion of the English leaves, and second to remember that it takes from 12—24 hours to act.

In regard to temperature, he did not think that high temperature of itself the most dangerous thing. In relapsing fever, for instance, the fever goes very high, and still the disease is not a fatal one. The same might be said of acute articular rheumatism.

Dr. Forchheimer did not have the confidence in digitalis that some of his friends had. He had used fresh infusion of the English leaves, and had been frequently disappointed. In these cases he used ergotin and tannic acid and had better results than when he used digitalis. The temperature is, he thought, looked upon as too important. It is only a symptom to the disease, and we may keep the temperature down and still have a fatal result from the violence of the poison.—*Cincinnati Lancet and Clinic.*

